# Parasitoid dynamics across decomposition stages in cadaver decomposition systems

**DOI:** 10.1093/jisesa/ieag067

**Published:** 2026-07-08

**Authors:** Nathalia Fonseca da Silva, Marcial Corrêa Cárcamo, Gabriele Maschke Jeske, João Luís Barbosa Marins Poulsen, Rodrigo Ferreira Krüger

**Affiliations:** Graduate Program in Animal Biodiversity (PPGBDiv), Institute of Biology, Federal University of Pelotas (UFPel), Pelotas, RS, Brazil; Laboratory of Parasite and Vector Ecology (LEPAV), Pelotas, RS, Brazil; Laboratory of Microbiology, Immunology and Parasitology (MIP), Federal Institute of Education, Science and Technology Sul-rio-grandense (IFSul), Pelotas—Visconde da Graça Campus, Pelotas, RS, Brazil; Laboratory of Microbiology, Immunology and Parasitology (MIP), Federal Institute of Education, Science and Technology Sul-rio-grandense (IFSul), Pelotas—Visconde da Graça Campus, Pelotas, RS, Brazil; Laboratory of Microbiology, Immunology and Parasitology (MIP), Federal Institute of Education, Science and Technology Sul-rio-grandense (IFSul), Pelotas—Visconde da Graça Campus, Pelotas, RS, Brazil; Laboratory of Parasite and Vector Ecology (LEPAV), Pelotas, RS, Brazil; Department of Microbiology and Parasitology, Institute of Biology, Federal University of Pelotas (UFPel), Pelotas, RS, Brazil

**Keywords:** cadaveric decomposition, forensic entomology, ecological succession

## Abstract

Parasitoid Hymenoptera associated with necrophagous Diptera represent a potentially valuable but underexplored component of decomposition systems and forensic entomology. This study investigated parasitoid species associated with pupae of *Chrysomya megacephala* (Fabricius, 1794) exposed during pig cadaver decomposition in the Pampa biome of southern Brazil, and evaluated parasitoid occurrence across decomposition stages and sampling periods. Experiments were conducted in March, September, and November 2024, and January 2025 using 12 pig cadavers. Sentinel pupae were exposed daily throughout decomposition and recovered for parasitoid emergence and identification. Generalized linear mixed models were used to evaluate parasitoidism probability and species-specific occurrence across decomposition stages, while Weibull survival models were applied to analyze stage duration. A total of 5,280 pupae were exposed, of which 376 (7.12%) were parasitized, yielding 4,171 parasitoid adults. Four species were recorded: *Nasonia vitripennis* (Walker, 1836) (Hymenoptera: Pteromalidae), *Pachycrepoideus vindemmiae* (Rondani 1875) (Hymenoptera: Pteromalidae), *Spalangia endius* Walker, 1839 (Hymenoptera: Spalangiidae), and *Tachinaephagus zealandicus* Ashmead, 1904 (Hymenoptera: Encyrtidae). Parasitoidism probability was significantly lower during the fresh stage and increased from the bloated stage onward. *Nasonia vitripennis* showed greater contribution during the bloated stage, whereas *P. vindemmiae* was proportionally more frequent during the fresh stage. Decomposition progressed more rapidly during warmer months, particularly in November and January. Seasonal variation in parasitoidism was also observed, with higher rates during warmer sampling periods. These findings demonstrate that parasitoid assemblages vary across decomposition stages and environmental conditions, highlighting their ecological relevance in decomposition systems and their potential contribution to forensic entomology in subtropical environments.

## Introduction

Decomposition systems are natural ecological processes driven by interactions between abiotic factors and diverse arthropod communities, particularly necrophagous insects and their natural enemies, which play a central role in nutrient recycling and ecosystem functioning (Sierra et al. 2017, Recinos-Aguilar et al. 2019). These communities follow predictable successional patterns, with Diptera typically colonizing cadavers early, followed by predators and parasitoids that exploit immature stages of these primary colonizers (Goff 2009, Amendt et al. 2011).

Within these systems, parasitoid Hymenoptera represent an ecologically relevant but still underexplored component (Amendt et al. 2000, Frederickx et al. 2013). By parasitizing dipteran larvae and pupae within specific temporal windows, these insects may function as “secondary clocks”, providing information even after fly emergence and extending the inferential window for postmortem interval (PMI) estimation in advanced forensic scenarios (Frederickx et al. 2013, Rivers 2016).

Despite this relevance, parasitoids remain underrepresented in forensic entomology studies, particularly regarding species-level identification and stage-specific associations (Amendt et al. 2000, Turchetto and Vanin 2004, Anderson et al. 2011, Horenstein and Salvo 2012, Sereno et al. 2016). As biological control of necrophagous flies involves both predators and parasitoids, the interaction between their life histories and the decomposition process may influence cadaveric fauna dynamics and, consequently, the accuracy of forensic estimations (Frederickx et al. 2013, Rivers 2016).

Among necrophagous Diptera, *Chrysomya megacephala* (Fabricius, 1794) is a widely distributed species of medical, veterinary, and forensic importance, characterized by rapid colonization and strong association with decomposing substrates (Badenhorst and Villet 2018, Cárcamo et al. 2021). Its pupae represent a key resource for parasitoid species, making it a suitable model for investigating parasitoid–host interactions under controlled conditions.

In this context, this study aimed to (i) describe the parasitoid species associated with *C. megacephala* in decomposition systems and their seasonal variation, and (ii) evaluate parasitoid occurrence across decomposition stages in the Pampa biome, southern Brazil.

## Methodology

### Study Site and Period

The study was conducted in a rural experimental area in southern Brazil (31°42′S; 52°18′W), within the Pampa biome. The Pampa is a subtropical grassland ecosystem characterized by extensive native grasslands, marked seasonal variation in temperature and precipitation, and a mosaic of natural and anthropogenic habitats associated with livestock production (Overbeck et al. 2007). The site comprises approximately 200 hectares and is characterized by livestock production systems and moderate anthropogenic activity.

Experiments were performed in March, September, and November 2024; and January 2025; using 3 cadavers per sampling month. Cadavers were positioned approximately 250 m apart to minimize overlap among insect assemblages associated with each cadaver, thereby reducing potential cross-attraction of colonizing insects and ensuring spatial independence among sampling units.

### Host Rearing and Sentinel Pupae

Pupae of *C. megacephala* were obtained from a laboratory colony obtained from adults collected on campus using funnel-type traps constructed from 2-L PET bottles and baited with chicken liver. Adults were maintained in 29-L ventilated containers and fed refined sugar, animal protein meal, and powdered milk (2:1:1), with water provided ad libitum, following the protocol adapted from Pires et al. (2009).

Colony conditions were maintained at 26 ± 2 °C, 70% ± 10% relative humidity, and a 12 h light:dark photoperiod. For oviposition, a substrate composed of animal protein meal, sawdust (2:1) and water was used to produce a paste-like medium, following Pires et al. (2010). Puparia used in the experiment were exposed within 24 h after pupariation.

### Experimental Design

Twelve stillborn pig cadavers (*Sus scrofa domesticus* L.), otherwise destined for sanitary disposal, were obtained from a commercial swine farm. Cadavers weighed between 1.73 and 2.10 kg and were stored frozen (−20 °C) until use. Before the experiment, cadavers were thawed for 12 h at ambient temperature in sealed plastic bags to prevent contamination.

Cadavers were placed individually in plastic containers (30 × 20 × 20 cm) on a 2-cm layer of moist sawdust. Containers were positioned approximately 250 m apart to ensure spatial independence and were not relocated during the experiment. The experimental setup was placed under roofed, open-sided shelters to reduce direct exposure to rainfall while maintaining natural environmental conditions and allowing insect access.

Cadavers were exposed to insect colonization for 72 h, a period considered sufficient for colonization by necrophagous dipteran species (Albushaabaa 2009, Essarras et al. 2021, Iancu et al. 2024), after which containers were covered with fine-mesh voile fabric (<1 mm aperture) to prevent further oviposition and colonization by necrophagous flies.

### Decomposition Monitoring and Sampling

Decomposition was recorded daily through photographic documentation and classified into stages (Fresh, Bloated, Active decay, Advanced decay, Skeletal) following Goff (2009).

After the initial 72 h, 20 pupae were exposed daily per replicate (60 pupae/day per month) until complete cadaver decomposition. Pupae were placed in polyethylene mesh bags positioned above the cadaver, preventing direct contact with decomposition fluids while allowing parasitoid access.

The use of sentinel pupae allowed standardization of host identity, pupal age, and exposure conditions across decomposition stages and sampling periods. This approach was adopted to specifically evaluate parasitoid species associated with *C. megacephala*, avoiding confounding effects caused by the simultaneous presence of naturally occurring dipteran hosts on cadavers. However, because sentinel pupae bypass natural oviposition and larval development processes, the resulting parasitoid assemblage reflects attraction to experimentally exposed pupae rather than naturally formed pupae within the decomposition substrate.

Collected pupae were transported to the laboratory, individualized in gelatin capsules (size 00), and maintained at 26 ± 2 °C, 70% ± 10% relative humidity, and a 12-h photoperiod. Pupae were monitored daily until emergence of flies or parasitoids.

Emerged adults were preserved in 70% ethanol and identified using standard taxonomic keys (Darling and Werren 1990, Froggatt 1921, Subba Rao 1978, Noyes et al. 1997, Gibson 2009). Pupae from which no emergence occurred after 90 d were dissected to assess the presence of parasitoids.

### Analyses

The parasitoidism rate was calculated as the proportion of parasitized pupae relative to the total number of exposed pupae, whereas parasitoid viability was calculated as the proportion of parasitized pupae from which adult parasitoids successfully emerged. These metrics were calculated for each sampling month and decomposition stage, allowing assessment of temporal and successional variation.

The probability of parasitoidism across decomposition stages was evaluated using a generalized linear mixed model (GLMM) with binomial error distribution, implemented with the ­glmmTMB package. The response variable was specified as a 2-column matrix of parasitized and non-parasitized pupae.

Decomposition stage and sampling month were included as fixed effects, whereas cadaver identity was included as a random intercept to account for repeated observations within the cadavers. Cadaver identity was defined as a unique combination of sampling month and replicate, reflecting the experimental structure composed of independent cadavers sampled within each month.

Model adequacy was assessed using simulated residual diagnostics (DHARMa). Estimated marginal means were obtained using the emmeans package, with Tukey-adjusted pairwise comparisons and 95% confidence intervals on the response scale.

Species-specific proportional contribution was analyzed using separate binomial GLMMs, considering only parasitized pupae. For each species, the response variable was defined as the number of pupae parasitized by that species relative to the total number of parasitized pupae within each observational unit.

*Tachinaephagus zealandicus* Ashmead, 1904 (Hymenoptera: Encyrtidae) was excluded from inferential analyses because of its low occurrence, which resulted in unstable model estimation, including convergence failures and Hessian singularity issues. Nevertheless, the species was retained in the descriptive analyses owing to its distinct biological strategy as a larval–prepupal parasitoid. In the models, decomposition stage was included as a fixed effect, whereas month was treated as a random effect.

The duration of decomposition stages was analyzed using parametric survival models assuming a Weibull distribution (survreg, package *survival*), appropriate for time-to-event data with non-constant hazard rates (Kelly and Smith 2011, Rehman et al. 2023). The response variable was the time (days) spent in each stage. Decomposition stage and sampling month were included as fixed effects, and cadaver identity was included as a clustering term to obtain robust standard errors. Model coefficients were exponentiated and interpreted as time ratios relative to reference categories.

Survival curves were derived from model predictions, and 95% confidence intervals were obtained by propagation of uncertainty from the variance–covariance matrix of the fitted model. All analyses were performed in R version 4.5.2 (R Core Team 2026).

Meteorological data used in the analyses were obtained from the Agromet platform (Embrapa Clima Temperado 2025), including monthly information on minimum, mean and maximum temperature, as well as relative humidity, during the study period.

## Results

A total of 5,280 pupae of *Chrysomya megacephala* were exposed, of which 376 (7.12%) were parasitized, yielding 4,171 adult parasitoids. The mean number of parasitoids per parasitized pupa was 11.1. However, brood size varied substantially among species in relation to their reproductive strategies.

Solitary parasitoids such as *P. vindemmiae* and *S. endius* produced approximately 1 adult per host, whereas the gregarious species *N. vitripennis* and *T. zealandicus* produced mean brood sizes of 15.0 and 18.0 individuals per parasitized pupa, respectively.

Parasitoidism rates varied across sampling months, with higher values recorded in January (19.16%) and November (12.25%) and lower values in September (1.38%) and March (1.52%) (Table 1).

**Table 1.** Number of exposed pupae, parasitized pupae, pupae with adult emergence, number of adult parasitoids, parasitoidism rate, and parasitoid viability rate, recorded in March, September, and November 2024, and January 2025 during the sampling period. The number of adult parasitoids reflects both parasitoidism frequency and brood size

Month	Number of exposed pupae	Number of parasitized pupae	Pupae with adult emergence	Number of adult parasitoids	Parasitoidism rate (%)	Parasitoid viability rate (%)
**March**	1,380	21	21	23	1.52	100
**September**	1,740	24	24	245	1.38	100
**November**	1,200	147	147	1,613	12.25	100
**January**	960	184	184	2,290	19.16	100

Four parasitoid species were recorded: *Nasonia vitripennis* (Walker, 1836) (Hymenoptera: Pteromalidae) represented 69.42% of the parasitized pupae records, followed by *Pachycrepoideus vindemmiae* (Rondani 1875) (Hymenoptera: Pteromalidae) (17.68%), *Spalangia endius* Walker, 1839 (Hymenoptera: Spalangiidae) (10.29%), and *Tachinaephagus zealandicus* Ashmead, 1904 (Hymenoptera: Encyrtidae) (2.11%) (Table 2).

**Table 2.** Relative contribution (%) of parasitoid species based on the number of parasitized pupae, by month and overall, during the 2024–2025 sampling period

Species	March (%)	September (%)	November (%)	January (%)	Total (%)
***Nasonia vitripennis* (Walker, 1836) (Hymenoptera: Pteromalidae)**	16.67	25.00	67.35	84.78	69.42
***Pachycrepoideus vindemmiae* (Rondani, 1875) (Hymenoptera: Pteromalidae)**	37.50	4.17	21.77	13.59	17.68
***Spalangia endius* Walker, 1839 (Hymenoptera: Spalangiidae)**	41.67	41.67	10.88	1.63	10.29
***Tachinaephagus zealandicus* (Ashmead, 1904) (Hymenoptera: Encyrtidae)**	4.17	29.17	0.00	0.00	2.11

**Fig. 1.**
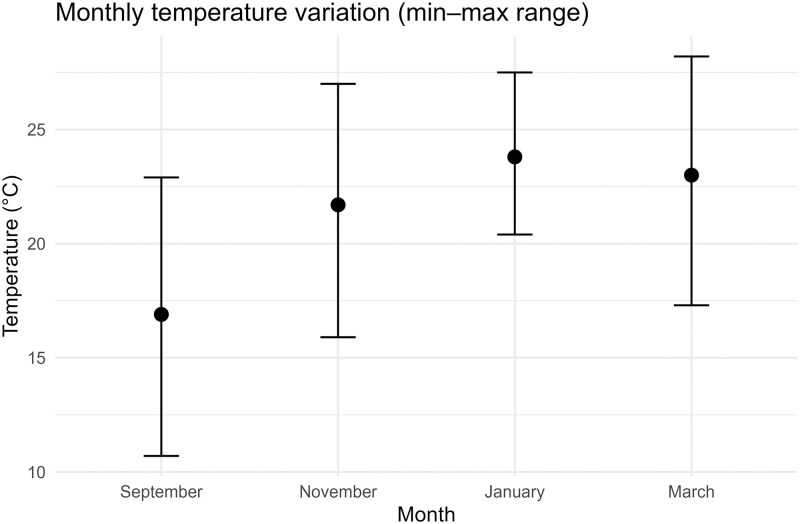
Monthly variation in temperature showing mean values (points) and minimum–maximum range (error bars) for March, September, and November 2024, and January 2025. Data obtained from Embrapa Clima Temperado.

Mean temperature ranged from 16.9 °C (September) to 23.8 °C (January), while relative humidity ranged from 82.3% to 90.3% (Fig. 1).

**Fig. 2.**
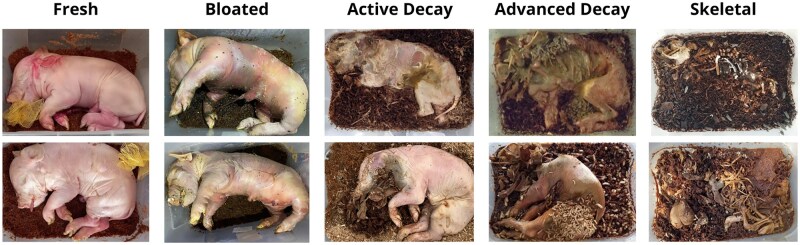
Representative images of pig carcasses at different decomposition stages (Fresh, Bloated, Active decay, Advanced decay, and Skeletal) following Goff (2009), observed across sampling months (March, September, and November 2024, and January 2025).

The duration of decomposition stages ranged from 1 to 16 days (Fig. 2). The Weibull survival model indicated significant effects of decomposition stage and sampling month on stage duration (χ^2^ = 39.56, df = 7, *P* < 0.001). Relative to the fresh stage, active decay (time ratio = 1.29; 95% CI: 1.23 to 1.35) and skeletal (4.24; 3.74 to 4.80) showed longer durations, whereas bloated (0.80; 0.59 to 1.08) and advanced decay (0.97; 0.70 to 1.34) did not differ significantly. Stage duration was shorter in November (0.32; 0.27 to 0.37), January (0.41; 0.37 to 0.45), and March (0.68; 0.56 to 0.82) compared to September (Table 3).

**Table 3.** Mean (±SD) duration (days) of decomposition stages across sampling months during the 2024–2025 period. Values represent descriptive statistics

Decomposition stage	March (mean ± SD)	September (mean ± SD)	November (mean ± SD)	January (mean ± SD)
**Fresh**	6.33 ± 1.15	4.00 ± 2.00	1.00 ± 0.00	2.00 ± 0.00
**Bloated**	3.33 ± 1.53	6.00 ± 4.36	1.00 ± 0.00	1.00 ± 0.00
**Active decay**	2.00 ± 0.00	8.00 ± 2.65	1.67 ± 0.58	4.33 ± 2.31
**Advanced decay**	3.67 ± 1.15	6.00 ± 2.00	1.00 ± 0.00	2.67 ± 1.53
**Skeletal**	7.67 ± 0.58	5.00 ± 5.20	15.30 ± 0.58	6.00 ± 1.00

The probability of parasitoidism varied significantly across decomposition stages. The GLMM showed that all stages from bloated onward had higher parasitoidism probability than the fresh stage (β = −5.39 ± 0.81, *P* < 0.001). Positive effects were observed for bloated (β = 2.41 ± 0.54), active decay (2.60 ± 0.52), advanced decay (2.79 ± 0.52), and skeletal (2.57 ± 0.51) (all *P* < 0.001). Estimated probabilities increased from 0.0045 (95% CI: 0.0009 to 0.0218) in Fresh to 0.0481 to 0.0691 in subsequent stages. Pairwise comparisons (Tukey-adjusted, response scale) indicated differences between Fresh and all other stages (*P* < 0.001), with no differences among later stages (*P* > 0.5).

The GLMM indicated significant effects of both decomposition stage and sampling month on parasitoidism probability. Relative to January, lower probabilities were observed in March (β = −2.58 ± 0.55, *P* < 0.001) and September (β = −2.98 ± 0.55, *P* < 0.001), whereas November did not differ significantly (β = −0.81 ± 0.51, *P* = 0.113). Model diagnostics based on simulated residuals indicated no major deviations from model assumptions and no evidence of significant overdispersion in the main GLMM (DHARMa dispersion test: dispersion = 1.75, *P* = 0.112). Convergence diagnostics indicated stable estimation for the primary models.

Species-specific models showed variation in relative contribution across stages. *N. vitripennis* had higher contribution in bloated compared to active decay (*P* = 0.0004) and skeletal (*P* < 0.0001). *P. vindemmiae* showed higher contribution in fresh compared to active decay (*P* = 0.0379) and skeletal (*P* = 0.0086). *S. endius* did not show significant differences among stages. *T. zealandicus* was excluded from statistical comparisons due to low frequency.

## Discussion

The parasitoid assemblage recorded in this study, composed of *N. vitripennis*, *P. vindemmiae*, *S. endius*, and *T. zealandicus*, is consistent with previous reports of parasitoids associated with necrophagous Diptera in decomposing substrates and anthropogenic environments (Whiting 1967, Marchiori 2005, Barbosa et al. 2008, Zhang et al. 2021, Silva et al. 2026). Although these species have been documented in decomposition studies across different regions (Early and Goff 1986, Anderson 1995, Marchiori et al. 2000, Voss et al. 2009, Frederickx et al. 2013), detailed resolution of their occurrence across decomposition stages remains limited in many systems.

The overall parasitoidism rate (7.12%) showed marked seasonal variation, with higher values in warmer months and reduced values during cooler periods. Temperature is frequently associated with increased parasitoid activity and development (Voss et al. 2010, Zhang et al. 2019), and the higher parasitoidism observed during November and January likely reflects more favorable environmental conditions for parasitoid foraging and development. However, because climatic variables were not explicitly modelled and environmental data were obtained from regional meteorological records rather than directly from cadaver microhabitats, the present results do not allow direct inference about the independent effects of temperature and humidity.

Although parasitoidism rates were lower than those reported in some decomposition studies (Voss et al. 2009) and other systems (Cárcamo et al. 2021, Silva et al. 2026), such differences are expected given variation in experimental design, cadaver type, exposure methodology, and regional environmental conditions.

*Nasonia vitripennis* was the dominant species throughout the sampling period, particularly during warmer months. This pattern is consistent with its high fecundity, rapid development under elevated temperatures, and broad host range (Rivers et al. 2012, Zhang et al. 2019). However, the ecological dominance of this species should be interpreted with caution, as its gregarious reproductive strategy and superparasitism behavior may substantially increase the number of emerging adults per host (King et al. 1995, Van Velzen et al. 2016). Consequently, the relative contribution based on parasitized pupae likely represents a more reliable ecological metric than absolute abundance alone.

*Pachycrepoideus vindemmiae* showed a relevant and less temporally concentrated contribution, suggesting broader ecological tolerance and the ability to exploit different temporal windows. *Spalangia endius* showed proportionally higher contribution in months with milder temperatures, such as September and March, which may reflect interspecific differences in foraging activity and performance under intermediate thermal conditions. In contrast, *Tachinaephagus zealandicus* occurred at low frequency, consistent with its biology as a gregarious endoparasitoid with an attack window associated with larval–prepupal stages (Voss et al. 2010). The scarcity of physiological and thermal data for some of these species under conditions comparable to those of the Pampa biome limits more detailed mechanistic interpretations and highlights the need for regional calibration.

Parasitoidism probability was minimal during the Fresh stage and increased significantly from the bloated stage onward. Because sentinel pupae remained continuously available throughout decomposition, this pattern likely reflects variation in parasitoid foraging activity and host-location efficiency rather than host availability itself. Progressive changes in decomposition-associated chemical cues may therefore influence parasitoid attraction and temporal species turnover (Frederickx et al. 2013).

Species-specific analyses also revealed distinct successional patterns. *Nasonia vitripennis* showed a greater contribution during the bloated stage, possibly reflecting its strong response to decomposition-associated odors (Mair and Ruther 2019). *Pachycrepoideus vindemmiae* showed a greater contribution during the fresh stage, suggesting earlier host-location ability or exploitation of microhabitats with reduced interspecific competition (Zhang et al. 2021, Manzano et al. 2023). In ­contrast, *Spalangia endius* showed no significant variation among stages, indicating a broader ecological tolerance. *Tachinaephagus zealandicus* remained rare throughout decomposition, consistent with its more restricted larval–prepupal attack window (Voss et al. 2010).

Because sentinel pupae remained continuously available throughout decomposition, the observed temporal variation among species likely reflects differences in behavioral responses to decomposition-mediated environmental and chemical gradients rather than host availability alone.

The duration of decomposition stages varied significantly among months and stages, with accelerated progression in warmer periods such as November and January. This result is consistent with the known influence of temperature on decomposition processes, microbial activity, and insect development (Voss et al. 2010, Zhang et al. 2019). Variation in stage duration may also influence parasitoid foraging dynamics by altering the temporal availability of hosts and the persistence of decomposition-associated chemical cues (Voss et al. 2009, Frederickx et al. 2013). Faster progression through early decomposition stages during warmer months may reduce the duration of resource windows associated with primary colonizers, whereas prolonged residual stages may extend opportunities for late-stage host exploitation and parasitoid activity.

An exception to this general pattern was observed for the skeletal stage in November, which showed longer duration than in the remaining sampling months. This may reflect rapid depletion of soft tissues during earlier stages, followed by a prolonged skeletal condition. Because the skeletal stage is strongly influenced by desiccation dynamics and residual tissue persistence (Payne 1965, Mann et al. 1990), environmental variation may substantially alter its temporal delimitation.

From a forensic perspective, the observed seasonal and successional patterns suggest that parasitoids may represent useful ecological indicators associated with later decomposition stages. Nevertheless, the present study should not be interpreted as direct validation of parasitoid-based PMI estimation under natural forensic conditions, particularly because sentinel pupae were used to standardize host availability and parasitoid exposure. In addition, parasitoidism itself may alter fly emergence dynamics and potentially bias PMI estimates based exclusively on dipteran development (Lin and Shiao 2013).

The marked variation observed among sampling months further suggests that regional environmental conditions may strongly influence parasitoid–host interactions in decomposing substrates. Given the pronounced seasonal variability characteristic of the Pampa biome (Overbeck et al. 2007), these findings emphasize the need for region-specific ecological knowledge when applying forensic entomology approaches in southern Brazil.
